# Targeting Cytokine Signals to Enhance γδT Cell-Based Cancer Immunotherapy

**DOI:** 10.3389/fimmu.2022.914839

**Published:** 2022-06-07

**Authors:** Yuan Song, Yonghao Liu, Huey Yee Teo, Haiyan Liu

**Affiliations:** ^1^ Immunology Programme, Life Sciences Institute, National University of Singapore, Singapore, Singapore; ^2^ Immunology Translational Research Program and Department of Microbiology and Immunology, Yong Loo Lin School of Medicine, National University of Singapore, Singapore, Singapore

**Keywords:** γδT cell, cytokine, cancer, immunotherapy, cellular therapy

## Abstract

γδT cells represent a small percentage of T cells in circulation but are found in large numbers in certain organs. They are considered to be innate immune cells that can exert cytotoxic functions on target cells without MHC restriction. Moreover, γδT cells contribute to adaptive immune response *via* regulating other immune cells. Under the influence of cytokines, γδT cells can be polarized to different subsets in the tumor microenvironment. In this review, we aimed to summarize the current understanding of antigen recognition by γδT cells, and the immune regulation mediated by γδT cells in the tumor microenvironment. More importantly, we depicted the polarization and plasticity of γδT cells in the presence of different cytokines and their combinations, which provided the basis for γδT cell-based cancer immunotherapy targeting cytokine signals.

## Characteristics and Antigen Recognition of γδT Cells

Although γδT cells share the same progenitors with conventional αβT cells and develop in the thymus, they are considered as innate immune cells due to their major histocompatibility complex (MHC) unrestricted antigen recognition, as well as the expressions of Natural Killer Receptors (NKRs) and Toll-like Receptors (TLRs) along with rapid cytokine production. The majority of γδT cells are negative for CD4 and CD8. In both human and mice, γδT cells account for 5% of total peripheral T cells.

**Figure 1:**
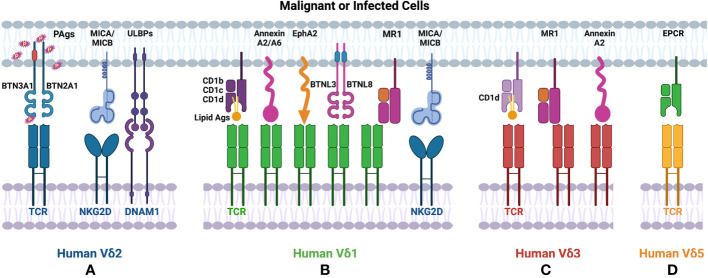
Ligands recognized by human γδT cells. **(A)** Human Vδ2 T cells recognize PAgs *via* TCR in a BTN molecule dependent manner. **(B)** TCRs of human Vδ1 cells recognize lipid antigens presented by CD1. Human Vδ1 also binds to Annexin A2/A6, EphA2, MR1 in an antigen-independent manner. **(A, B)** Both human Vδ1 and Vδ2 T cells express NKRs (such as NKG2D, DNAM1), which bind to MICA/MICB, ULBPs expressed on tumor cells. **(C)** Human Vδ3 cells interact with CD1d with/without antigen via TCR, also recognize Annexin A2 or MR1 without antigen loading. **(D)** Human Vd5 cells bind to EPCR *via* TCR.

Based on the TCR δ chain usage, human γδT cells can be subtyped to Vδ1, Vδ2, Vδ3 and Vδ5 cells (**Table 1**). Vδ1 and Vδ2 are the major subsets, which are of great interest among human γδT cells. Human Vδ2 cells are generally paired with T cell receptor (TCR) γ9, also named as Vγ9Vδ2 cells. Vγ9Vδ2 cells are the dominant γδT subset in human peripheral blood mononuclear cells (PBMCs). Vγ9Vδ2 TCRs recognize phosphoantigens (PAgs) such as isopentenyl pyrophosphate (IPP), which is accumulated in tumor cells, and (E)-4-hydroxy-3- methyl-but-2-enyl pyrophosphate (HMBPP) that is produced during microbial infections (**Figure 1A**). Interestingly, although γδT cells bind PAgs in the MHC independent manner, PAgs-mediated activation of Vγ9Vδ2 requires butyrophilin (BTN) and BTN-like molecules (1). Recent studies reported that BTN2A1 associated with BTN3A1 to initiate antigen-presentation to Vγ9Vδ2 T cells (2, 3). Besides TCR-associated antigen recognition, Vγ9Vδ2 T cells also express NK receptors including NKG2D and DNAM1, which recognize MHC class I chain-related molecules (MICA, MICB), ULBP-binding proteins (ULBPs) and Nectin-like-5 that are broadly expressed on tumor cells (4).

**Table 1:** γδT subsets and distribution in human and mouse.

Species	δ Chain	γ Chain	Distribution
human	Vδ1	Vγ2, Vγ3, Vγ4, Vγ5, Vγ8, and Vγ9	dermis, gut, thymus, liver, and other epithelial tissues, PB
	Vδ2	Vγ9, Vγ8, Vγ4	PB, liver
	Vδ3	various γ chains	liver, gut, PB
	Vδ5	Vγ4	PB
mouse	Vγ1	high diversity	spleen, blood, lymph node, liver, lung, dermis
	Vγ4	high diversity	spleen, blood, lymph node, liver, lung, dermis
	Vγ5	Vδ1	dermis
	Vγ6	Vδ1, Vδ4	reproductive mucosa, skin
	Vγ7	Vδ4, Vδ5, Vδ6	gut

Human Vδ1 cells are mainly distributed in epithelial tissues, such as skin, gut, spleen, and liver. Human Vδ1 cells constitute only up to 15% of human γδT cells in PBMCs (5), but they exhibit fast and marked expansion during CMV infections (6). γδT cell compartment involved in HCMV-specific response is non-Vγ9Vδ2 T cells with the TCRVδ1^+^ lymphocytes representing the prominent non-Vγ9Vδ2 γδ T cell subset (7–9). Furthermore, these Vδ1 cells display a mixed CD27^-^/CD45RA^+^ or CD27^+^/CD45RA^+^ phenotype that is identified as cytotoxic effector/memory populations in CMV^+^ individuals (10). These findings indicated the potential immune surveillance function of Vδ1 cells. Whereas the TCRγ chains paired with Vδ1 display high diversity and the antigens recognized by Vδ1 cells are not well revealed, it has been shown that CD1 molecules with or without loaded lipid antigens can specifically activate Vδ1 cells. The direct interactions between Vδ1 and CD1b, CD1c, or CD1d have been identified by CD1 tetramers, mutagenesis experiments and crystal structures (11–15). In addition to CD1-associated recognition, Vγ4Vδ1 cells have been reported to respond to BTNL3 and BTNL8 expressing cells *via* Vγ4 chain (**Figure 1B**) (16). Annexin A2 and Annexin A6 that are known as stress-induced phospholipid-binding proteins and involved in tumorigenesis also stimulated the proliferation and the production of TNF-α in Vγ4Vδ1 cells (17). Another newly identified stress-induced antigen that is recognized by Vδ1 TCR is ephrin type-A receptor 2 (EphA2) (**Figure 1B**), which is upregulated upon AMP-activated protein kinase (AMPK)-dependent metabolic reprogramming of cancer cells. It can be recognized co-ordinately by ephrin A to govern the activation of Vγ9Vδ1 cells (18). The involvement of EphA2 in Vδ1-mediated tumor cell lysis was demonstrated by reduced susceptibility to killing by EphA2 blocking (19). Human Vδ1 cells from peripheral blood and tissues exhibit autoreactivity to the monomorphic MHC-related protein 1 (MR1) without binding with any ligands, indicating MR1 as a ligand of Vδ1 γδTCR (20). Similar to Vδ2 cells, Vδ1 cells also mediate tumor cell lysis through recognizing ULBP3 and MICA by NKG2D (**Figures 1A, B**) (21–23).

Vδ3 cells account for ~0.2% of lymphocytes in PBMCs from healthy donors but are enriched in the liver and gut and can be expanded in patients with CMV activation and B cell chronic lymphocytic leukemia (24, 25). Human Vδ3 cells were identified as CD1d-restricted T cells and can mediate specific killing against CD1d^+^ cells (**Figure 1C**). Different from Vδ1 cells, Vδ3 cells can not recognize other CD1 molecules (such as CD1b,CD1c) (26). Annexin A2 was identified as the direct ligand of Vγ8Vδ3 TCR (**Figure 1C**) (17). Recently, human Vδ3 cells have also been shown to bind to MR1 in an antigen-independent manner (**Figure 1C**). Another notable population of human γδT cells is Vδ5 subset. Human Vγ4Vδ5 T cells were reported to bind directly with endothelial protein C receptor (EPCR) (**Figure 1D**), which is a MHC-like molecule and binds to phospholipid (27). However, the phospholipid binding is not required for the recognition between human Vδ5 cells and EPCR (28).

Taken together, in contrast to αβT cells and other unconventional T cells, such as NKT and MAIT cells, human γδT cells usually recognize specific molecules in an antigen-independent manner except for Vδ2 cells. For example, Vδ1 and Vδ3 TCRs bind to the underside of MR1 and the side of the MR1 antigen-binding groove respectively. Vδ1 cells also respond to CD1 without the loading of lipid antigens. Vδ5 cells recognize EPCR without the involvement of antigens. Other than the recognition of these MHC-like structures in the absence of antigens, Vδ1 TCR can also interact with Annexin A2 and A6 and Vδ3 TCR can recognize Annexin A2 in an Ig-like manner.

With regard to murine γδT cells, they are generally grouped by the usage of TCR γ chains (**Table 1**). Vγ1 and Vγ4 are the predominant subsets in the splenic and circulating γδT cells (29). They are located in many mouse tissues. Vγ5 is invariably paired with Vδ1 and the Vγ5Vδ1 cells are found in dermis and are also named as dendritic epidermal T cells (DETC) (30). Vγ6 cells are mainly paired with Vδ1 or Vδ4 and can home to the mucosa of reproductive tissues and skin (30–32). Vγ7 cells are restricted to intestinal epithelial lymphocytes (33). However, the recognition of PAgs of γδTCR was not found in mouse. Only limited studies reported antigens recognized by murine γδT cells, such as H2−T10, H2−T22, and algae protein phycoerythrin (PE) (34–37). A recent study found that BTNL molecules shape the local Vγ7 and Vγ5 compartments in murine intestinal epithelium and skin (16, 38). The requirement of BTNL during the selection and maintenance of tissue-resident γδT cells indicates the potential interaction between γδTCR and BTNL. However, it is still not clear how the murine and human γδT cell subsets can be matched with each other, and it is difficult to translate some of the findings with murine γδT cells directly to human.

## Anti-Tumor and Pro-Tumor Functions of γδT Cells Mediated by Cytokines and Receptor-Ligand Interactions

**Figure 2:**
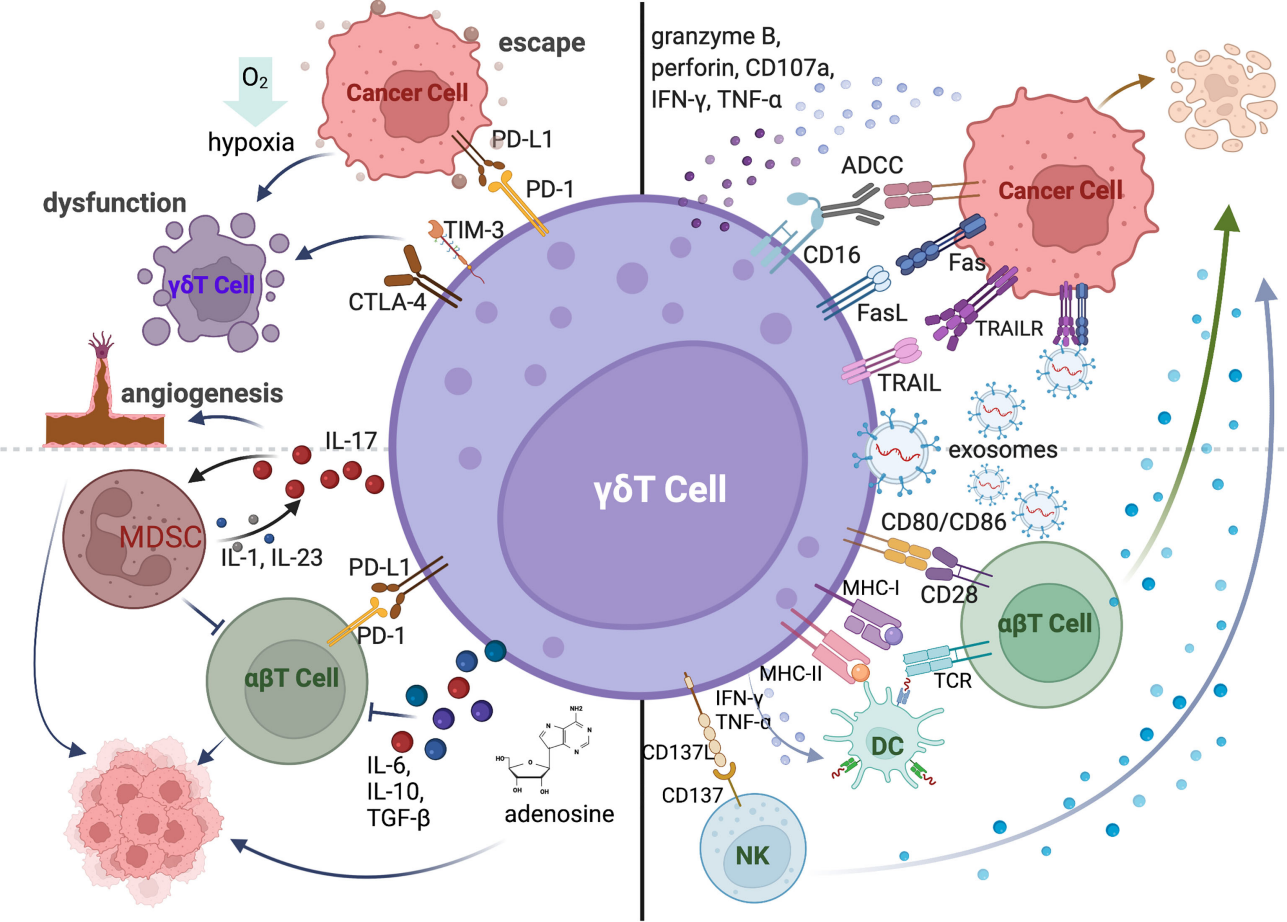
The anti-tumor and pro-tumor functions of γδT cells mediated by cytokines and receptor-ligand interactions. γδT cells can directly kill tumor cells by expressing death receptor ligands (FasL, TRAIL), producing cytotoxic molecules (granzyme B, perforin, CD107a, IFN-γ and TNF-α) and mediating ADCC *via* CD16 expression. The exosomes derived from γδT cells can also directly induce the apoptosis of cancer cells. γδT-APC can activate conventional T cells *via* MHC-I, MHC-II, and co-stimulatory molecules. γδT cells induce the maturation of DCs by secreting IFN-γ and TNF-α and trigger the activation of NK cells *via* CD137L. The pro-tumor function of γδT cells is mediated by the expression of co-inhibitory receptors. The co-inhibitory molecules contribute to tumor cell escape from immune surveillance. Hypoxic tumor microenvironment also induces the dysfunction of γδT cells. γδT cells also promote the tumor growth by recruiting immunosuppressive cells and inhibiting conventional T cells *via* producing IL-17, IL-6, IL-10, TGF-β or adenosine.

After the recognition of antigens or other stress-induced molecules expressed on tumor cells by TCR or NKR, γδT cells can mediate the direct tumor lysis by producing granzyme B, perforin, TNF-α and IFN-γ (**Figure 2**: top right) (39, 40). For example, human Vγ9Vδ2 T cells induced human hepatocellular carcinoma cell lysis in a DNAM-1-dependent manner (4). IL-17 produced by γδT17 cells significantly inhibited tumor development in mice and patients with lung cancer (41, 42). Additionally, activated γδT cells also express death induced ligands CD95L (also known as FasL) and TNF-related apoptosis-inducing ligand (TRAIL), which engage with death receptor CD95 (Fas) and TRAIL receptor, and apoptosis of infected or malignant cells (43–45). Similar to NK cells, the majority of γδT cells in peripheral blood express CD16. CD16 acts as an activation site triggering antibody dependent cellular cytotoxicity (ADCC) (**Figure 2**: top right) (46). A recent study showed that exosomes derived from human Vγ9Vδ2T cells (γδT-Exos) efficiently induced the apoptosis of tumor cells through death receptor ligation (**Figure 2**: top right) (47, 48).

In addition to the direct killing against tumor cells, γδT cells can exert the indirect anti-tumor function by regulating other immune cells in the tumor microenvironment (**Figure 2**: bottom right). Human Vδ2 T cells are described as professional antigen-presenting cells, which can process antigens and provide co-stimulatory signals to induce the proliferation and differentiation of αβT cells (49). It is also reported that human γδT-APCs efficiently cross-present soluble antigens to CD8^+^T cells *via* MHC-I (50, 51). The high expression levels of APC-associated molecules and tumor antigen presenting capability of *in vitro* expanded human Vγ9Vδ2 T cells were also detected during the early stage of differentiation (52). Activated human γδT cells boost NK cell mediated killing of tumor cells through CD137L (53).

Besides ligand-receptor interactions, cytokine production is the pivotal pathway to regulate other immune cells. Like conventional T cells, γδT cells can be polarized to different subsets based on the secreted cytokines, including IFN-γ-producing γδT cells (γδT-IFN or γδT1), IL-4-producing γδT cells (γδT2), IL-17-producing γδT cells (γδT17) and Foxp3^+^ regulatory γδT cells (γδTreg). These cytokine-producing γδT cells exist in both human and mouse and can regulate other immune cell functions *via* their signature cytokine productions (**Figure 2**: bottom right). For instance, activated γδT1 cells promoted the maturation of DCs *via* IFN-γ dependent manner in mouse (54). Human freshly isolated γδT1 cells also induced the upregulation of HLA-DR, CD86, CD83 and release of IFN-γ, IL-6, and TNF-α of monocyte-derived DCs through the production of TNF-α and IFN-γ (55, 56). Both human Vδ2 and Vδ3 cells can promote B cell differentiation, antibody maturation and cytokine production (25, 55). IL-4 producing mouse Vγ1Vδ6 T cells can drive the proliferation and IgA secretion of Germinal Centre (GC) B cells (57). In additions, γδT17 cells promoted the infiltration of CTLs within the tumor bed *via* IL-17 production after chemotherapy (58).

Although the anti-tumor functions of γδT cells have been shown in many murine models and in cancer patients, the pro-tumor activities of γδT cells were also reported in numerous studies (**Figure 2**: left). Co-inhibitory molecules can be upregulated on human and murine γδT cells in tumors, which can bind to the co-inhibitory receptors expressed on αβT cells to restrain their activation, infiltration, and anti-tumor efficiency (59). The expressions of PD-1, TIM3 and TIGIT also induced the exhaustion and dysfunction of γδT cells in AML and MM patients (60). Moreover, co-inhibitory receptors on γδT cells contribute to the tumor immune escape by interaction with immunosuppressive molecules (**Figure 2**: top left) (61). Meanwhile, hypoxic tumor microenvironment induced by metabolic status of cancer cells is a critical factor in mediating immunosuppression. The anti-tumor function of γδT cells can be inhibited by hypoxia *via* the downregulation of NKG2D and CD107a expressions (62, 63). Over the past decade, IL-17-producing γδT cells have been found to associate with enhanced tumor growth and metastasis. γδT is one of the major sources of IL-17 in the tumor microenvironment and reduced tumor burden was observed in IL-17-producing Vγ4-depleted and IL-17-deficient mice (64). γδT17 cells recruit myeloid-derived suppressor cells (MDSCs) to the tumor site, which can suppress CD8^+^T cell responses (64, 65). Consistently, this is also demonstrated in human colorectal cancer (66). In addition, IL-17-produing γδT cells can accelerate tumor progression by promoting angiogenesis and mobilizing pro-tumor macrophages (67, 68). γδTreg cells were found to impair DC maturation and function and CD8^+^T cell-mediated anti-tumor function in cancer patients *via* TGF-β, IL-6 or IL-10 dependent or independent manner (69, 70). Moreover, CD39^+^ γδTregs were implicated in the immunosuppressive environment *via* producing adenosine in human colorectal cancer (71). The IL-6-adenosine positive feedback loop between CD73^+^ γδTregs and cancer-associated fibroblast (CAF) was also involved in tumor progression in breast cancer patients (72).

The role of γδT cells during tumor development is still controversial. Their functions could be cancer type specific. For example, human Vδ1 cells exhibit potent cytotoxicity against colon cancer cells and B-cell chronic lymphocytic leukemia (73, 74), whereas Vδ2 cells are shown to kill a wide variety of tumors including acute myeloid leukemia, multiple myeloma and lung cancer (60, 75). On the other hand, some γδT subsets may exert different functions in the same type of cancer under different treatment conditions/environment. γδT17 cells promoted CTL infiltration into colon cancer after chemotherapy (58), whereas they have been reported to inhibit anti-tumor immune response *via* promoting the recruitment, proliferation, and survival of MDSCs in colorectal cancer and hepatocellular carcinoma (66). Therefore, γδT cell function during tumor development may be greatly influenced by the cytokines present in the tumor microenvironment under specific conditions.

## Cytokine-Mediated Regulation of γδT Cell Function

**Figure 3:**
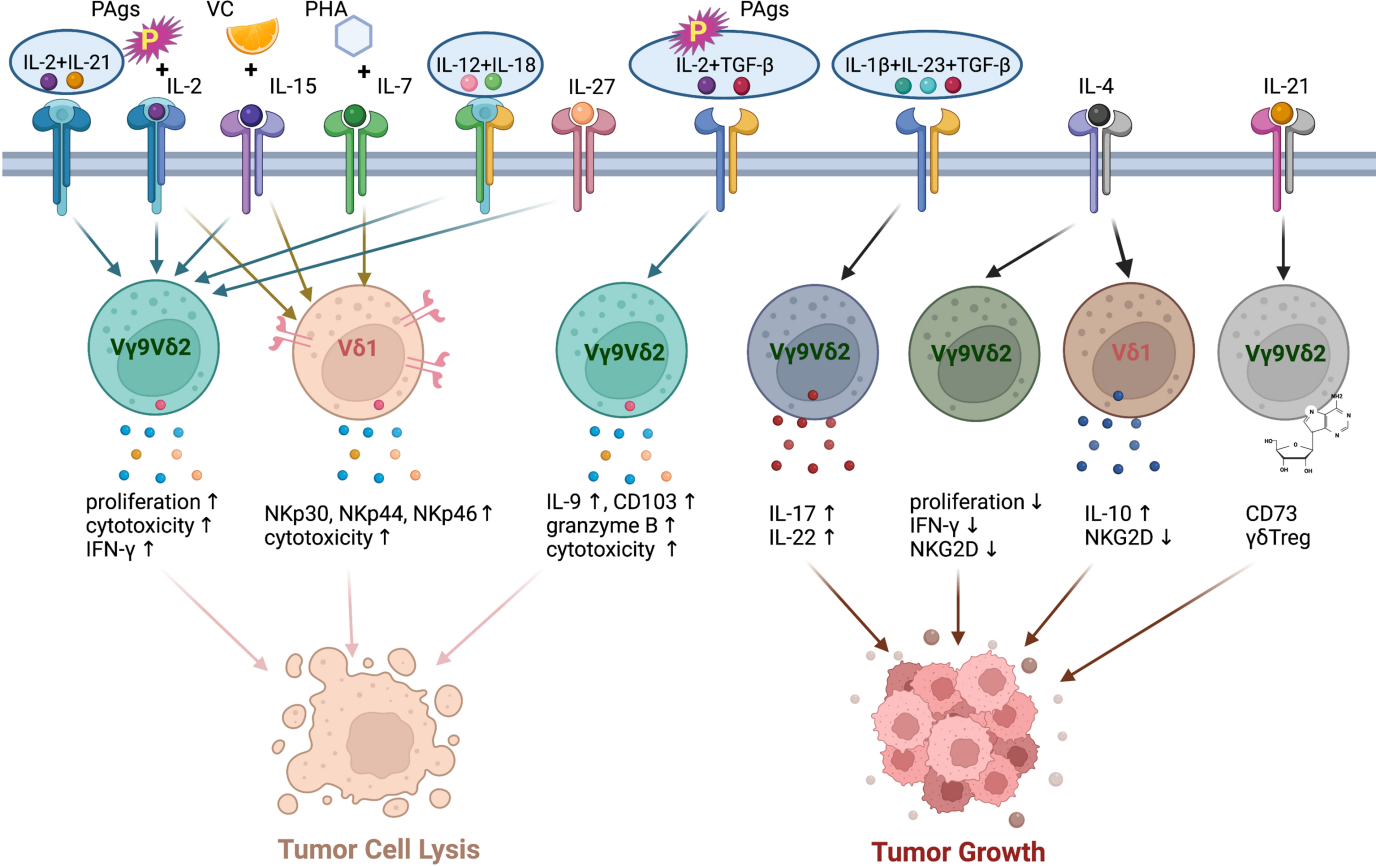
The polarization of human γδT cells induced by different cytokine combination. PAgs and IL-2 with the addition of VC and IL-15, IL-12+IL-18, IL-27, IL-21+IL-2 enhance the cytotoxicity of human Vγ9Vδ2 T cells. IL-2 or IL-15 induces the expressions of NKp30, NKp44, NKp46 on human Vδ1 cells. PHA and IL-7 enhance the cytotoxic capacity of human Vδ1 cells. TGF-β increases the anti-tumor cytotoxicity of human Vγ9Vδ2 T cells in the presence of PAgs and IL-2. The combination of IL-1β, IL-23 and TGF-β promotes the differentiation of Vγ9Vδ2 T cells to IL-17-producing γδT cells. IL-4 reduces the proliferation, NKG2D expression and IFN-γ production of Vγ9Vδ2 T cells *via* promoting IL-10 secretion of Vδ1 cells. IL-21 alone induces Vγ9Vδ2 T cell differentiation to CD73^+^ γδTreg cells, which promote tumor growth.

IL-2 is the commonly used cytokine for expanding human and murine γδT cells. IL-2 is identified as T cell growth factor and is necessary for the proliferation and differentiation of naïve T cells into effector T cells (76). However, γδ T cells produce relatively less IL-2 than αβ T cells (77). Due to the PAgs recognition of human Vγ9Vδ2 T cells, the combination of IL-2 with synthetic PAgs, such as Zoledronate (Zol) and BrHPP, was widely used for the generation of human Vγ9Vδ2 T cells from PBMCs for γδT cell-based immunotherapy (**Figure 3**). Adoptive transfer of pamidronate-expanded Vγ9Vδ2 cells alone effectively prevented EBV-induced B cell lymphoproliferative disease (EBV-LPD) in mouse and the injection of pamidronate significantly controlled the development through specific activation and expansion of Vγ9Vδ2 cells in humanized mice (78). The adoptive transfer of IL-2/PAgs *ex vivo* expanded Vγ9Vδ2 cells from autologous or allogeneic hosts exhibited potent anti-tumor effects in a variety of cancer patients, such as gastric cancer, osteolytic breast cancer, prostate cancer, and colorectal cancer and so on (79–81). The *in vivo* administration of pamidronate/Zol and low-dose IL-2 also triggered the proliferation of γδT cells in clinical trials and engaged the anti-tumor response without appreciable toxicity in patients (82–84).

IL-15, another proinflammatory cytokine in IL-2 superfamily, has been shown to contribute to the effector functions and maintain the survival of human NK cells *via* IL-15-AKT-XBP1s signalling pathway (**Figure 3**) (85). It is also a promising candidate for enhancing the expansion and cytotoxicity of γδT cells. With the stimulation of IL-2 or IL-15, human Vδ1 cells were selectively induced to express NKp30, NKp44 and NKp46 in a PI3K/AKT dependent manner. The expression of NCRs is associated with increased production of granzyme B and improved cytotoxicity against tumor cells (86, 87). Although low IL-2 and additional IL-15 did not affect NKR expression level on human Vγ9Vδ2 cells, IL-15 significantly increased the expressions of perforin, granzyme B, granulysin and T-bet, which led to enhanced cytotoxic capacity of Vγ9Vδ2 cells. A recent study showed that IL-15 and vitamin C (VC) promoted the proliferation and differentiation and reduced the apoptosis of human Vγ9Vδ2 T cells *in vitro* (88). Moreover, these cells possessed improved cytotoxicity, both *in vitro* and in humanized mouse model. The adoptive transfer of IL-15+VC expanded Vγ9Vδ2 T cells prolonged the survival of patients with late-stage lung cancer or liver cancer (89). IL-15 receptor α signalling limited the development of IL-17-produing γδT cells in a mouse model (90). A global increase of γδT17 cells was found in IL-15Rα-KO mice, but only modest dysregulation of IL-17 production was observed on γδT cells from IL-15-KO mice (90).

Other members of IL-2 cytokine family, including IL-4, IL-7, and IL-21, can also act on γδT cells (**Figure 3**). IL-4 was demonstrated to negatively regulate the anti-tumor function of γδT cells *via* inhibiting the expression of NKG2D and promoting the IL-10 production from Vδ1 cells, which in turn suppressed IFN-γ production and the proliferation of Vδ2 cells (91). IL-7 was used to expand Vδ1 cells from PBMCs in the presence of PHA *in vitro*. These expanded Vδ1 cells exhibited great anti-tumor function and prolonged the survival of human colon carcinoma xenografted mice *via* expressing high levels of cytotoxicity-related molecules, chemokine receptors and NCRs (73). However, IL-7 selectively promoted the IL-17 production of human Vδ1, Vδ2 from cord blood and murine CD27- γδT cells (92). The combination of IL-2 and IL-21 directly enhanced the cytotoxicity of human γδT cells to hepatocellular carcinoma cells *in vitro* (93). In the presence of IL-21, PAgs-expanded Vγ9Vδ2 T cells expressed high level of CXCR5, which enhanced their potential to support antibody production by B cells (94). On the other hand, IL-21-stimulated Vγ9Vδ2 T cells can differentiate to CD73^+^ γδTreg cells, which exert immunosuppressive function *via* inhibiting T cell responses (95).

The synergistic function of IL-12 and IL-18 in inducing the IFN-γ production of T cells and NK cells has been demonstrated (96–99). Similarly, IL-12 and IL-18 also induced the production of IFN-γ and increased cytotoxicity in γδT cells in an antigen-independent manner (**Figure 3**) (100, 101). However, the combination of IL-12 and IL-18 led to the upregulation of TIM3 on γδT cells (102). That might indicate the exhaustion or dysfunction of γδT cell under the treatment of IL-12/18. IL-27 is a heterodimeric cytokine of IL-12 cytokine family. The expression of IL-12R on T cells can be induced by IL-27 (103). The expression of IL-27R was also detected on human Vγ9Vδ2 cells. As expected, IL-27 enhanced the cytotoxicity of human Vγ9Vδ2 T cells by promoting the production of cytotoxic molecules (**Figure 3**) (104).

In addition to cytokines inducing IFN-γ production in γδT cells, IL-17-inducing cytokines are responsible for the polarization of γδT17 cells. It is well known that combination of IL-1β, IL-6, IL-23 and TGF-β induce Th17 differentiation in mouse (105). In human, IL-1 and IL-23 but not TGF-β and IL-6 serve as a rheostat tuning the magnitude of Th17 development (106). The stimulation of IL-1 and IL-23 also promoted RORγt, IL-17, IL-21, and IL-22 expression by γδT cells without the engagement of T cell receptor in mouse (107, 108). TGF-β was found to play a key role in the generation of murine γδT17 in thymus during the postnatal period (109). In adults, IL-1β, TGF-β and IL-23 are required for the commitment of human Vγ9Vδ2 T cells to IL-17-producing γδT cells, which also produce IL-22 (110). The function of IL-6 during the differentiation of γδT17 is uncertain. However, the cocktail of cytokines (IL-1β, TGF-β, IL-6 and IL-23) was used to selectively generate IL-17^+^ Vγ9Vδ2 T cells *in vitro* (111). These expanded IL-17^+^ Vγ9Vδ2 T cells produce IL-17 but neither IL-22 nor IFN-γ. The expressions of granzyme B, TRAIL, FasL and CD161 on IL-17^+^ Vγ9Vδ2 T cells indicated that they contributed to host immune responses against infectious microorganisms. By contrast, TGF-β surprisingly augmented the cytotoxic activity of human Vδ2 T cells when they were stimulated with PAgs and IL-2 or IL-15 in the presence of TGF-β. TGF-β enhanced the migration and anti-tumor function of Vδ2 T cells through upregulating the expressions of CD54, CD103, IFN-γ, IL-9 and granzyme B (112, 113).

In conclusion, γδT cells display high functional plasticity depending on the cytokine environment (**Figure 3**). In view of the cytokine-dependent polarization of γδT cells, it is crucial to understand the roles of various cytokines regulating γδT cell function, which can guide the effective γδT cell-based cancer immunotherapy.

## Current γδT Cell-Based Cancer Immunotherapies

**Figure 4:**
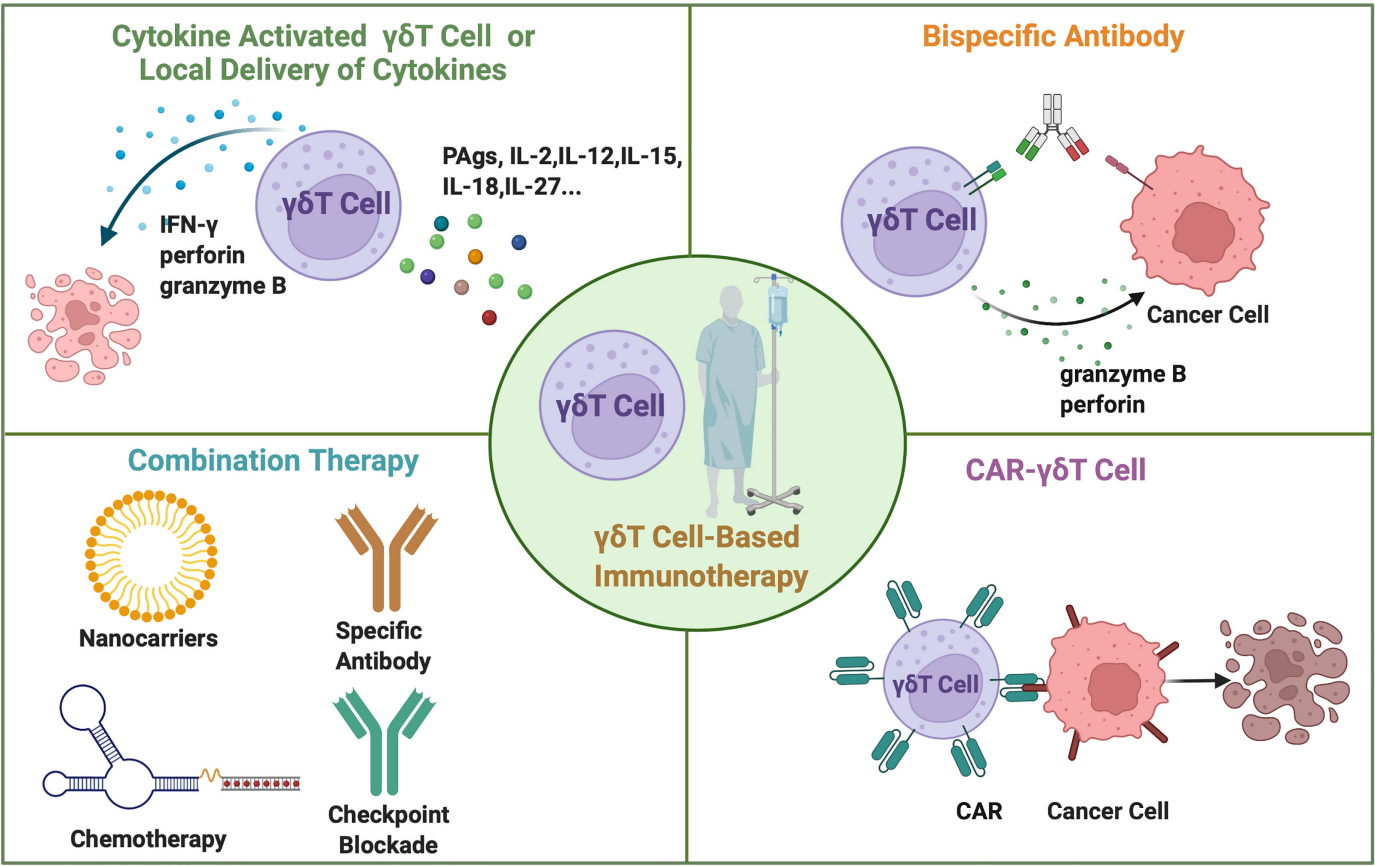
The current approaches for γδT cell-based cancer immunotherapy. The adoptive transfer of cytokine-activated γδT cells *in vitro* or locally administration of cytokines *in vivo*. Combination therapy includes γδT cell transfer combined with specific antibody therapy, immune checkpoint blockade, chemotherapy, and nanoparticles. Bispecific antibodies simultaneously bind to γδT cells and cancer cells. Gene modified CAR-γδT cells directly recognize the cancer cells and mediate cancer cell lysis.

Currently, the majority of the preclinical and clinical studies on γδT cell-based cancer immunotherapy focus on adoptive transfer of expanded γδT cells and its combination with other treatments (**Table 2**, **Figure 4**: top left and bottom left). Due to the feasible expansion of human Vγ9Vδ2 T cells using PAgs or aminobisphosphonates, Zol has been used to expand human γδT cells for adoptive transfer or directly injected to induce the proliferation of human γδT cells *in vivo* for cancer immunotherapy (115, 136).

**Table 2:** Clinical trials of γδT cell-based immunotherapy.

Cell types	Cancer type	Phase	Stimulation	Ref
Both Vδ1 and Vδ2 cells	Lymphoma	I	Anti-γδ T-cell receptor (TCR) antibody combine with IL-2 *in vitro* expanded	(114)
Vγ9Vδ2	Renal cell carcinoma	I/II	Zoledronate and IL-2 *in vivo*	(115)
Vγ9Vδ2	Renal cell carcinoma, Colon cancer, Oesophagus carcinoma, Gastric cancer, Ovarian cancer, Breast cancer	I	Bromohydrin pyrophosphate (IPH1101) combine with IL-2 *in vivo*	(116)
Vγ9Vδ2	Metastatic renal cell carcinoma	I	Bromohydrin pyrophosphate (IPH1101) combine with IL-2 *in vivo*	(117)
Vγ9Vδ2	Non-Hodgkin lymphoma (NHL) or Multiple myeloma (MM)	Pilot study	IL-2 combine with pamidronate	(84)
Vγ9Vδ2	Renal cell carcinoma	Pilot study	IL-2 *in vivo*	(118)
Vγ9Vδ2	Breast cancer	II	Neoadjuvant letrozole (LET) plus zoledronic acid	(119)
Vγ9Vδ2	Colorectal cancer	Unknown	Zoledronate and IL-2 *in vitro* expansion	(120)
Vγ9Vδ2	Myeloma	II	Zoledronate and IL-2 *in vivo*	(121)
Vγ9Vδ2	Neuroblastoma	I	Zoledronate and IL-2 *in vivo*	(82)
Vγ9Vδ2	Leukaemia	Pilot study	Zoledronate and IL-2 *in vivo*	(122)
Vγ9Vδ2	Renal cell carcinoma [RCC], Malignant melanoma, and Acute myeloid leukemia	I/II	Zoledronate and IL-2 *in vivo*	(123)
Vγ9Vδ2	Renal cell carcinoma	Pilot study	Zoledronate and IL-2 *in vivo*	(124)
Vγ9Vδ2	Breast cancer	II	zoledronic acid *in vivo*	(125)
Vγ9Vδ2	Non-small cell lung cancer	I	Zoledronate and IL-2 *in vitro* expansion	(126)
Vγ9Vδ2	Non-small cell lung cancer	I	Zoledronate and IL-2 *in vitro* expansion	(127)
Vγ9Vδ2	Breast cancer	I	Zoledronate and IL-2 *in vivo*	(128)
Vγ9Vδ2	Various solid tumors	Unknown	zoledronic acid *in vitro*	(129)
Vγ9Vδ2	Breast cancer	Unknown	zoledronic acid *in vivo*	(130)
Vγ9Vδ3	Multiple myeloma	Pilot study	Zoledronate and IL-2 *in vitro* expansion	(131)
γδ T	Pancreatic cancer	I	Combination of gemcitabine (GEM) and autologous γδ T-cell therapy	(132)
γδ T	Locally advanced pancreatic cancer	II	Irreversible electroporation plus allogeneic γδ T cells	(133)
γδ T	Hepatocellular carcinoma (HCC) and intrahepatic cholangiocarcinoma (ICC).	I/II	Locoregional therapy followed by adoptive transfer of allogeneic γδ T cells	(134)
γδ T	Non-muscle invasive bladder cancer	II	Rapamycin and BCG instillations	(135)

Due to the successful application of chimeric antigen receptor (CAR) technology in αβT cells, it has also been applied in γδT cell therapy (**Figure 4**: bottom right). The study of allogeneic CAR-Vδ1 T cells targeting CD20 antigen exhibited strong anti-tumor activity and minimum xenogeneic graft-versus-host diseases (GVHD) post transplantation (137). This result further supports the clinical evaluation of ADI-001, an allogeneic CD20-CAR-Vδ1 T cell-associated clinical trial (NCT04735471). CAR-Vδ2 T cells also showed promising results in clearing tumor *in vivo* (138). Mucin 1 (MUC1) with the Tn epitope is a tumor associated antigen that is highly expressed on the surface of a variety of cancer cells. MUC1-Tn CAR-modified Vγ9Vδ2 T cells exhibited similar or stronger anti-tumor effect against breast cancer cell and gastric cancer cell *in vitro* compared with CAR-αβT cells. MUC1-Tn-CAR-Vγ9Vδ2 T cells more effectively suppressed tumor growth than Vγ9Vδ2 T cells in a xenograft murine gastric cancer model (138).

Many recent studies focus on antibody-induced γδ T cell activation (**Figure 4**: top right). Fab fragment of anti-CD3e antibody UCHT1 could bind to γδTCR and enhance the tumor killing of Vγ9Vδ2 T cells (139). Aude De Gassart et al. constructed a humanized antibody, ICT01, that could activate Vγ9Vδ2T cells (140). This antibody activated γδT cells that could kill various tumor cell lines and primary tumor cells but not normal healthy cells. Rajkumar Ganesan et al. designed a bispecific antibody, anti-TRGV9/anti-CD123, that could simultaneously bind to the Vγ9 chain of Vγ9Vδ2 T cells and AML target antigen, CD123, then induce the recruitment and activation of Vγ9Vδ2 T cells to target AML blasts (141). Recently, it is demonstrated that tribody activated γδT cells efficiently. Hans H Oberg et al. reported that tribody [(HER2)2 X CD16] is more effective than anti-HER2 monoclonal antibodies in enhancing γδT cell killing against HER2-expressing cancer cells (142). Similarly, tribody of (Her2)2X Vγ9 targets human Vγ9 T cells and HER2-expressing tumor cells to induce γδT cell-mediated tumor killing (143).

The combination therapy of γδT cells with chemotherapy, monoclonal antibody, immune checkpoint blockade or surgery can exert better anti-tumor efficacy than monotherapy (**Figure 4**: bottom left). The combination of γδT cells with locoregional therapy enhanced clinical efficacy (134). The study using rituximab combined with obinutuzumab and daratumumab activated γδT cells expanded the therapeutic potential of distinctive tumor-antigen-targeting mAbs induced ADCC by γδT cells (144). Targeting the costimulatory signals such as CD137 agonist antibody may promote the anti-tumor functions of Vγ9Vδ2 T cells (145). γδT cell therapy enhanced chemotherapy-induced cytotoxicity to advanced bladder cancer cells (146). Chemotherapeutic agent temozolomide (TMZ) may promote the anti-tumor efficacy of the adoptively transferred *ex vivo* expanded γδT cells for malignant glioblastoma (147). A few studies demonstrated that nanoparticles could also enhance γδ T cells function. In a recent work, it was found that selenium nanoparticles (SeNPs) pre-treatment strengthened the anti-tumor cytotoxicity of Vγ9Vδ2 T cells by increasing the expression of cytotoxicity related molecules, such as NKG2D, CD16, and IFN-γ (148). Chitosan nanoparticles (CSNPs) also exhibited the role of enhancing anti-tumor immune responses of γδT cells (149). Immune checkpoint blockade using anti-PD-1 mAb promoted Vγ9Vδ2 T cell cytotoxicity against PC-2 tumors in immunodeficient NSG mice (150). Furthermore, combination of Tim-3 blocking antibody and bispecific antibody MT110 (anti-CD3 and anti-EpCAM) enhanced the anti-tumor efficacy of the adoptively transferred γδT cells (151). However, autologous γδT cells combined with gemcitabine therapy for patients with curatively resected pancreatic cancer revealed no significant difference compared with those receiving gemcitabine alone (132), suggesting better understanding of the mechanism of action during different treatment is required to achieved effective combination treatment outcome with γδT cells. Cytokine combinations promoting γδT cell function revealed in pre-clinical studies are yet to be evaluated in clinical trials.

## Challenges and Potential Strategies Targeting Cytokine Signals to Improve γδT Cell-Based Immunotherapy

γδT cell-based immunotherapy mainly faces three challenges in achieving improved outcomes for cancer patients. The first challenge is the *in vitro* generation/expansion of activated γδT cells with superior cytotoxicity. Although adoptive transfer or *in vivo* expanded human Vγ9Vδ2 T cells exhibited good safety profile, it did not achieve clinical benefit in some patients (123). To boost the cytotoxicity of expanded γδT cells and overcome the immune suppressive tumor microenvironment, cytokine stimulated allogeneic Vγ9Vδ2 cells or Vδ1 cells have been used for clinical trials. A recent study on 132 late-stage cancer patients confirmed the safety and efficacy of IL-15 and VC activated allogeneic Vγ9Vδ2 T cells (89). The addition of IL-15 resulted in the activation, proliferation and increased cytotoxic capacity of γδ T cells (152). To activate cytokine signals, expanded Vδ1 T cells were engineered with a GPC-3 CAR and secreted IL-15 (sIL-15) which significantly controlled tumor growth without inducing GVHD. Moreover, GPC-3-CAR/sIL-15 Vδ1 T cells displayed greater proliferation and stronger anti-tumor responses when compared with GPC-3-CAR Vδ1 T cells lacking sIL-15, suggesting IL-15 signal was critical for CAR Vδ1 T cell function (153). The adoptive transfer of IL-7-expanded human Vδ1 cells also displayed improved cytotoxicity and prolonged the survival of human colon carcinoma xenografted mice (73).

Secondly, rapid exhaustion is a big challenge for maintaining survival and durable anti-tumor functions of γδT cells. Persistent stimulation of human γδT cells with PAgs often induces γδT cell exhaustion (154). It was demonstrated that CD137 costimulation promoted the proliferation and prolonged the survival of Vγ9Vδ2 T cells *in vitro* and *in vivo* (145). Moreover, Endogenous IL-15 acted as a potential factor to support the survival of human Vγ9Vδ2 T cells *in vivo* in the absence of exogenous IL-2 (120). The dysfunction of T cells is also associated with the immunosuppressive tumor microenvironment which will be discussed in the following session.

The third challenge is the immunosuppression mechanisms in cancer patients that can impair the anti-tumor functions of the infused/activated γδT cells. The lack of IL-2 and IL-21 in HCC patients was associated with the PD-1 expression and reduced cytotoxicity of human γδT cells (93). In a murine HCC model, IL-23 overexpression in the liver induced the polarization of γδT cells to IL-17-producing γδT cells (155). Then γδT17 cells promoted tumor growth *via* recruiting immunosuppressive myeloid-derived suppressor cells (MDSCs). TGF-β is a pivotal immunosuppressive cytokine that secreted by immunosuppressive cell subsets (such as MDSCs and Treg) and tumor cells (156). Mouse Foxp3^+^ γδT cells can be induced by TGF-β and inhibit T cell activation (157). To avoid γδT cell exhaustion and circumvent tumour immunosuppressive microenvironment, it is a feasible approach to target cytokine signals *via* administering exogenous stimulating cytokines or blocking the immunosuppressive cytokines. As systemic administration of cytokines usually induces toxicity in patients (158, 159), local delivery of cytokine can limit the systemic toxicity and offer an approach to benefit from the therapeutic effects of the activating cytokines. The local delivery of mRNAs encoding interleukin-12 (IL-12) single chain, interferon-α, granulocyte-macrophage colony-stimulating factor, or IL-15 sushi led to robust anti-tumor immune responses and tumor regression in multiple murine models (160). These findings provided preclinical evidence for modifying the tumor microenvironment *via* local administration of cytokines. It is possible to induce highly cytotoxic γδT cells through modulations of tumor microenvironment through the induction or delivery of cytokines that can specially promote the anti-tumor functions of γδT cells (**Figure 4**: top left).

## Conclusion and Future Directions

Taken together, γδT cells are promising cellular products for adoptive cancer immunotherapy. γδT cells mediate anti-tumor effects by direct killing and indirect immune regulatory function to other immune cells. γδTCR can recognize specific molecules often in an antigen-independent manner. γδT cells can differentiate into various subsets producing signature cytokines, which can have anti-tumor or pro-tumor functions. In the meantime, this differentiation is greatly influenced by the cytokines present in the microenvironment. γδT cell-based cancer immunotherapy has a good safety profile in the clinical trials but its clinical efficacy needs further improvement. Combination therapies involving γδT cells have had some clinical successes, including chemotherapy, CAR therapy, and checkpoint blockade therapy. IL-2 and IL-15 have been explored for their functions to activate γδT cells in clinical trials. However, other cytokines and combinations that can activate γδT cells are yet to be evaluated in clinical trials. First, cytokines or cytokine combinations can be used to expand, activate, and polarize γδT cells *ex vivo* to generate potent cellular products for adoptive therapy. Cytokine signals can also be modulated to prolong the survival of the transferred γδT cells *in vivo*. Second, cytokine can be incorporated into CAR γδT cell therapy to facilitate CAR γδT cell function and prolong their survival *in vivo via* autocrine mechanism, which can avoid the toxicity induced by systemic cytokine treatment. Third, cytokine signal on γδT cells can be triggered *via* antibody binding in the form of bi-specific or tri-specific antibody targeting tumor antigens. The additional cytokine signal can facilitate γδT cell function and survival. Thus, detailed understanding of the effects of cytokines and cytokine combinations on γδT cell anti-tumor function is critical for designing effective therapeutic strategies to incorporate cytokine signals into various γδT cell-based cancer immunotherapy to achieve superior clinical efficacy.

## Author Contributions

All authors listed have made a substantial, direct, and intellectual contribution to the work and approved it for publication.

## Funding

This work has been supported by Singapore National Research Foundation grant NRF-CRP19-2017-04.

## Conflict of Interest

The authors declare that the research was conducted in the absence of any commercial or financial relationships that could be construed as a potential conflict of interest.

## Publisher’s Note

All claims expressed in this article are solely those of the authors and do not necessarily represent those of their affiliated organizations, or those of the publisher, the editors and the reviewers. Any product that may be evaluated in this article, or claim that may be made by its manufacturer, is not guaranteed or endorsed by the publisher.
